# Improving glycemic control: transitioning from dulaglutide to tirzepatide in patients with type 2 diabetes undergoing hemodialysis

**DOI:** 10.3389/fphar.2024.1362242

**Published:** 2024-05-30

**Authors:** Emiko Otsuka, Mineaki Kitamura, Satoshi Funakoshi, Hiroshi Mukae, Tomoya Nishino

**Affiliations:** ^1^ Department of Nephrology, Nagasaki University Graduate School of Biomedical Sciences, Nagasaki, Japan; ^2^ Nagasaki Renal Center, Nagasaki, Japan; ^3^ Department of Respiratory Medicine, Nagasaki University Graduate School of Biomedical Sciences, Nagasaki, Japan

**Keywords:** tirzepatide, dulaglutide, hemodialysis, continuous glucose monitoring, GLP-1, GIP, CGM, glycemic control

## Abstract

**Background:** Tirzepatide—a dual glucose-dependent insulinotropic peptide and glucagon-like peptide-1 receptor agonist—is used to treat type 2 diabetes. However, the efficacy and safety of tirzepatide in patients undergoing hemodialysis remain unclear.

**Methods:** We conducted a single-center retrospective study of patients with type 2 diabetes undergoing hemodialysis who were transitioned from dulaglutide to tirzepatide. We continuously monitored glucose levels in patients undergoing hemodialysis before and after switching from dulaglutide to tirzepatide.

**Results:** Fourteen patients (mean age: 61.9 ± 9.9 years, male: female = 11:3) were included in this study. After switching to tirzepatide, time in range increased to 50.8% from 42.7% (*p* = 0.02), time above range decreased to 37.8% from 48.4% (*p* = 0.02), and mean glucose levels decreased to 137.4 mg/dL from 156.6 mg/dL (*p* = 0.006). In contrast, there was no significant difference in time below range before and after tirzepatide administration (11.3% and 8.9%) (*p* = 0.75). Three patients experienced dyspepsia (21.4%), and one patient experienced nausea (7.1%); however, no critical adverse events were reported.

**Conclusion:** Transitioning from dulaglutide to tirzepatide improved glycemic control without increasing hypoglycemia in patients undergoing hemodialysis for type 2 diabetes.

## 1 Introduction

Diabetes is the most common cause of end-stage renal disease (ESRD). Patients with type 2 diabetes have a high mortality rate, mainly owing to cardiovascular diseases. This is particularly evident in patients undergoing hemodialysis (Saran et al., 2017; Ahmadmehrabi and Tang, 2018). Glycemic control is crucial to improve clinical outcomes and significantly reduce cardiovascular risk and mortality. However, anti-diabetic drugs such as sodium-glucose cotransporter 2 (SGLT2) inhibitors may be of limited use or contraindicated in ESRD (Rocco and Berns, 2012; Williams and Garg, 2014); thus, they are often treated with insulin. Glycemic control with insulin is often difficult, especially in patients undergoing hemodialysis. Insulin is partially metabolized in the kidneys, and its effects are prolonged in patients with renal insufficiency (Rave et al., 2001). Blood insulin and glucose levels fluctuate during hemodialysis and even after dialysis (Abe et al., 2007). Therefore, patients undergoing hemodialysis frequently experience large glycemic excursions that put them at a greater risk of hyperglycemia and hypoglycemia (Galindo et al., 2023).

Incretin hormones are currently commonly used for the treatment of diabetes. The main incretin hormones are glucagon-like peptide-1 (GLP-1) and glucose-dependent insulinotropic peptide (GIP) (Nauck and Meier, 2018).

Dulaglutide, a weekly formulation of the GLP-1 receptor agonists (GLP-1 RA) launched in Japan in 2015, is effective in reducing body weight, controlling fasting and postprandial glycemia, and has a positive effect on atherosclerotic cardiovascular outcomes (Ruda et al., 2023). The current guidelines for managing type 2 diabetes recommend GLP-1 RAs as first-line injectable therapy even before insulin initiation (American Diabetes Association Professional Practice Committee, 2022). Moreover, dulaglutide has also been reported to be useful in glycemic control in patients undergoing hemodialysis (Yajima et al., 2018; Ugamura et al., 2022). However, some patients treated with dulaglutide have inadequate glycemic control and need more effective drugs.

**FIGURE 1:**
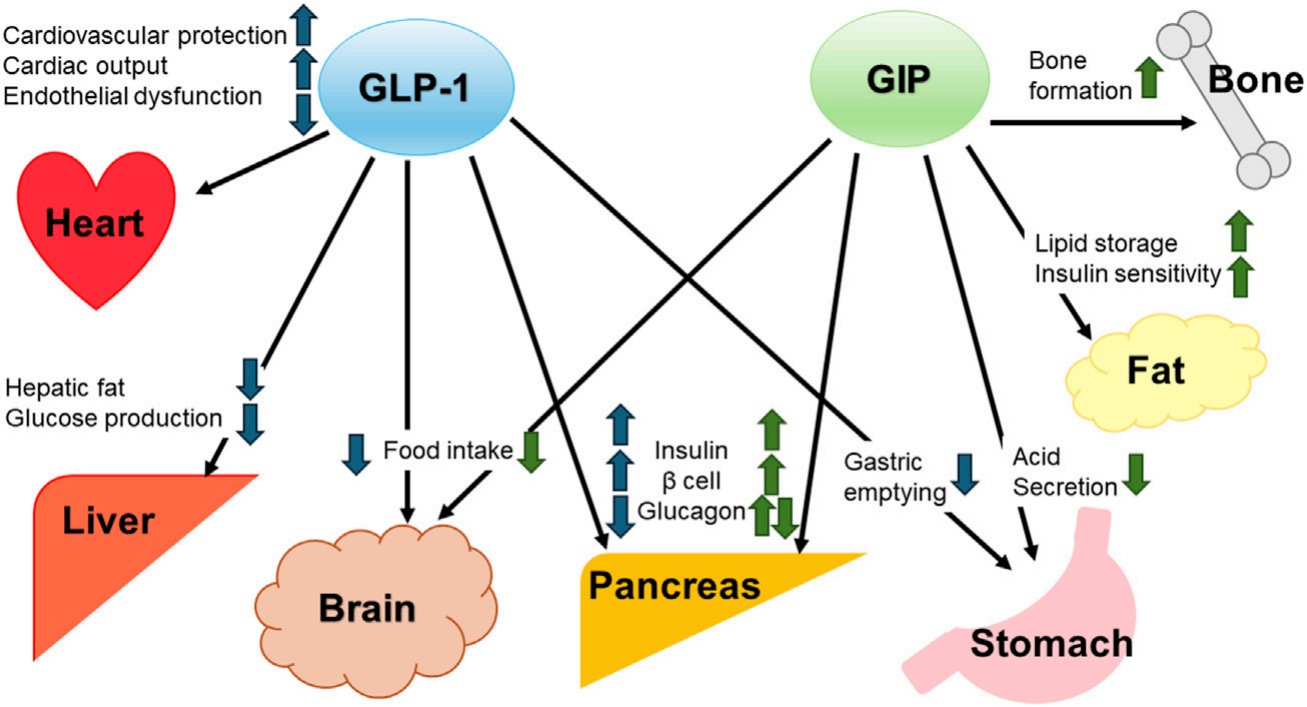
Complementary effects of GIP and GLP-1 GIP, glucose-dependent insulinotropic peptide. GLP-1, glucagon-like peptide-1.

Tirzepatide is a novel dual GLP-1 and GIP agonist (Coskun et al., 2018). A combination of GLP-1 and GIP can act on pancreatic beta cells synergistically and complementarily through distinct metabolic effects (Bastin and Andreelli, 2019). Moreover, GIP exerts therapeutic benefits beyond its primary incretin role by improving insulin sensitivity and lipid homeostasis in adipose tissue (Asmar et al., 2016). GLP-1 and GIP delay gastrointestinal secretion and suppress appetite, resulting in more effective glycemic control and body weight reduction (Holst, 2021). In addition, GLP-1 is reported to be cardioprotective (Mosenzon et al., 2021) and hepatoprotective (Muzurović et al., 2022), and GIP is reported to inhibit bone resorption (Nissen et al., 2014). The complementary effects of GIP and GLP-1 are shown in Figure 1. Tirzepatide significantly improves glycemic control in patients with type 2 diabetes with or without basal insulin regimens (Dahl et al., 2022; Karagiannis et al., 2022), and a clinical trial demonstrated the superiority of tirzepatide compared with dulaglutide in terms of glycemic control (Inagaki et al., 2022).

However, the safety and efficacy of tirzepatide in patients undergoing hemodialysis remain unclear. Therefore, we aimed to compare the glycemic control between dulaglutide and tirzepatide in patients undergoing hemodialysis. We analyzed the transition in glucose levels using continuous glucose monitoring (CGM) before and after switching from dulaglutide to tirzepatide in patients undergoing hemodialysis.

## 2 Materials and methods

### 2.1 Study design and patients

We included patients with type 2 diabetes undergoing hemodialysis whose prescriptions for diabetes were transitioned from dulaglutide to tirzepatide at the Nagasaki Renal Center between June 2023 and August 2023. Blood glucose levels were analyzed using CGM. Patients who changed their insulin dose or other diabetic drugs during this period were excluded.

### 2.2 Blood glucose measurement

Blood glucose levels were evaluated using CGM for 7 days in patients on dulaglutide and tirzepatide. Dulaglutide was dosed at 0.75 mg once a week for at least 3 months and CGM was started during the hemodialysis sessions and recorded for 7 days. After the discontinuation of CGM, tirzepatide (2.5 mg) was administered 1 week after the last dose of dulaglutide. The second CGM period started 7–14 days after initiating tirzepatide treatment and was recorded for 7 days. All patients undergoing hemodialysis at our facility did not eat during the dialysis sessions to avoid hypotension. However, the included patients were asked to eat as usual outside dialysis hours. Consequently, each CGM period included 3 days of hemodialysis days and 4 days of non-hemodialysis days. Mean blood glucose levels and time in range (TIR), time above range (TAR), and time below range (TBR) were analyzed using FreeStyle Libre Pro (Abbott Japan Tokyo, Japan).

We set the TIR at 80–140 mg/dL, TAR at >140 mg/dL, and TBR at <80 mg/dL. An international expert panel published a consensus on the glycemic target range of 70–180 mg/dL and a percentage of reading time per day of over 70% (Danne et al., 2017; Battelino et al., 2019). According to the Internal Consensus on the Use of CGM 2017, 70–140 mg/dL is a secondary target range (Danne et al., 2017), and some studies show that this tight range improves the survival rate in critically ill patients (Krinsley and Preiser, 2015; Lanspa et al., 2019). Moreover, a target range of 70–140 mg/dL seems to have advantages over a 70–180 mg/dL range for assessing glycemic status and progress toward stricter glycemic control, particularly when approaching normal glucose levels (Dunn et al., 2024). However, it is crucial to prevent excessive hypoglycemia in patients with severe complications such as renal failure. Therefore, we set the threshold at 80 mg/dL.

### 2.3 Statistical analysis

Values are shown as the mean ± standard deviation. Differences in TIR, TAR, TBR, and mean blood glucose levels before and after switching to tirzepatide were analyzed using the Wilcoxon signed-rank test. A *p*-value of <0.05 was considered statistically significant. Statistical analyses were conducted using JMP Pro 17.0.0 (SAS Institute Inc., Cary, NC, United States).

### 2.4 Ethical statements

This study was approved by the Clinical Research Ethics Committee of the Nagasaki Renal Center (Nagasaki, Japan) (approval number: 23025) and was conducted in accordance with the 1964 Declaration of Helsinki and its subsequent amendments. The requirement for informed consent was waived by the Clinical Research Ethics Committee of the Nagasaki Renal Center (Nagasaki, Japan) owing to the retrospective study design.

## 3 Results

Sixteen patients with type 2 diabetes undergoing hemodialysis were transitioned from dulaglutide to tirzepatide at the Nagasaki Renal Center from June 2023 to August 2023. All the participants were prescribed 0.75 mg of dulaglutide once a week for at least 3 months and transitioned to 2.5 mg of tirzepatide once a week. Two patients were excluded because their insulin dose or other anti-diabetic drugs were changed during the study period. One patient was hospitalized owing to an infection, and his insulin dose decreased due to appetite loss during hospitalization. Another patient was prescribed liraglutide outside the facility.

Fourteen patients without other diabetes medications or insulin changes during the observation period were included in this study. All patients underwent hemodialysis three times a week for 4 h in each dialysis session. Their mean age was 61.9 ± 9.9 years; 11 were male and 3 were female. The cause of ESRD was type 2 diabetes in 13 patients and chronic glomerulonephritis in one patient. The duration of diabetes was unknown for four patients, while the mean disease duration for the other 10 patients was 25.7 ± 7.5 years, with 45.4 ± 32.2 (months) under dulaglutide. The baseline patient characteristics and data after transitioning from dulaglutide to tirzepatide are shown in Table 1. Interdialytic weight gain and total protein declined after transitioning from dulaglutide to tirzepatide; however, albumin and Geriatric Nutritional Risk Index did not show significant changes.

**TABLE 1:** Baseline patient characteristics and data after transitioning from dulaglutide to tirzepatide.

Characteristic	Baseline	After transitioning	*p*-value
Age (years)	61.9 ± 9.9	—	—
Sex (male) number (%)	11 (78.6)	—	—
Dialysis vintage (years)	5.6 ± 2.5	—	—
Diabetes vintage (years)	25.7 ± 7.5	—	—
Dosing dulaglutide (months)	45.4 ± 32.2	—	—
Dry weight (kg)	71.6 ± 17.1	71.5 ± 16.9	0.75
BMI (kg/m^2^)	25.7 ± 6.7	25.1 ± 6.5	0.73
IDWG (kg)	3.5 ± 1.2	2.7 ± 1.6	0.02
Glycated Albumin (%)	21.4 ± 3.6	21 ± 4.7	0.45
Hemoglobin (g/dL)	11.0 ± 1.06	10.8 ± 0.9	0.55
Creatinine (mg/dL)	10.4 ± 1.6	10.8 ± 2.1	0.17
BUN (mg/dL)	56.7 ± 14.7	53.6 ± 15.5	0.22
Total protein (g/dL)	6.8 ± 0.6	6.6 ± 0.5	0.04
Albumin (g/dL)	3.6 ± 0.4	3.5 ± 0.4	0.19
Potassium (mEq/L)	5.1 ± 0.6	5.3 ± 1.4	0.35
Calcium (mg/dL)	8.7 ± 0.4	8.7 ± 0.5	0.98
Phosphate (mg/dL)	5.5 ± 1.6	5.3 ± 1.4	0.35
GNRI	94.0 ± 6.1	92.6 ± 6.5	0.12
Insulin treatment number (%)	5 (35.7)	5 (35.7)	—
Oral anti-diabetic agent treatment			
Glinide number (%)	3 (21.4)	3 (21.4)	—
Alpha-glucosidase inhibitor number (%)	3 (21.4)	3 (21.4)	—

Data are expressed as the median ±SD. Insulin treatment and oral anti-diabetic agent treatment data are expressed as the number of patients (%).

BMI, body mass index; IDWG, interdialytic weight gain; BUN, blood urea nitrogen; GNRI, geriatric nutritional risk index.

**FIGURE 2:**
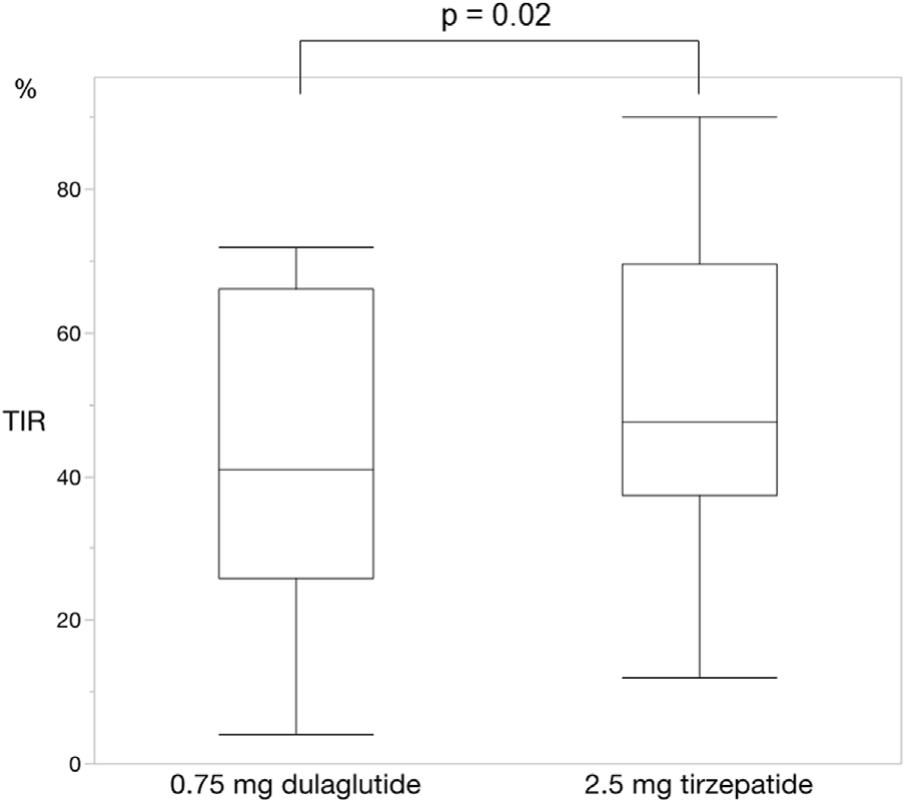
Change in time in range. The time in range significantly improved changing from dulaglutide to tirzepatide. Wilcoxon rank-sum test was used in the analysis. TIR, time in range.

**FIGURE 3:**
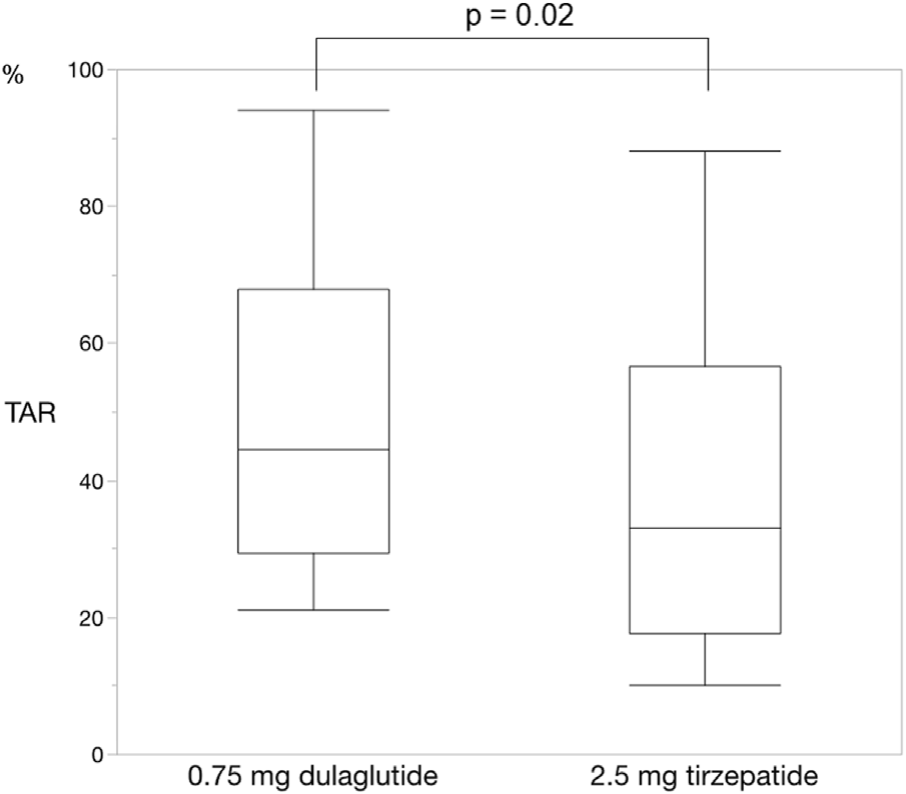
Change in time above range. The time above range also improved changing from dulaglutide to tirzepatide. Wilcoxon rank-sum test was used in the analysis. TAR, time above range.

**FIGURE 4:**
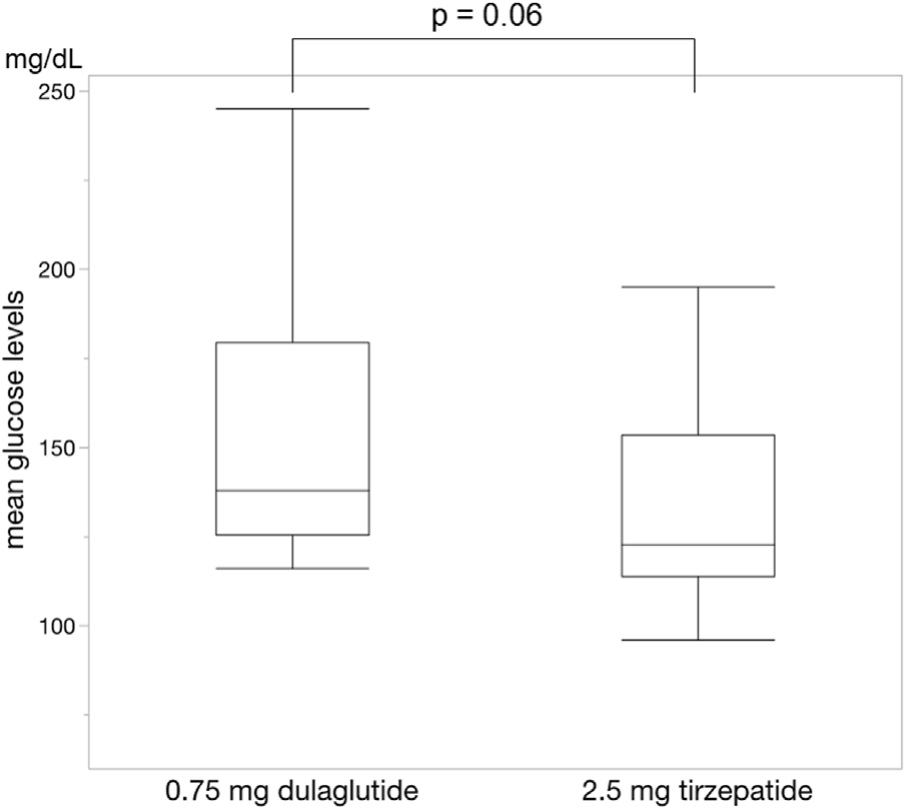
Change in mean blood glucose levels. The mean blood glucose levels were significantly lower during administrating tirzepatide than that of dulaglutide. Wilcoxon rank-sum test was used in the analysis.

**FIGURE 5:**
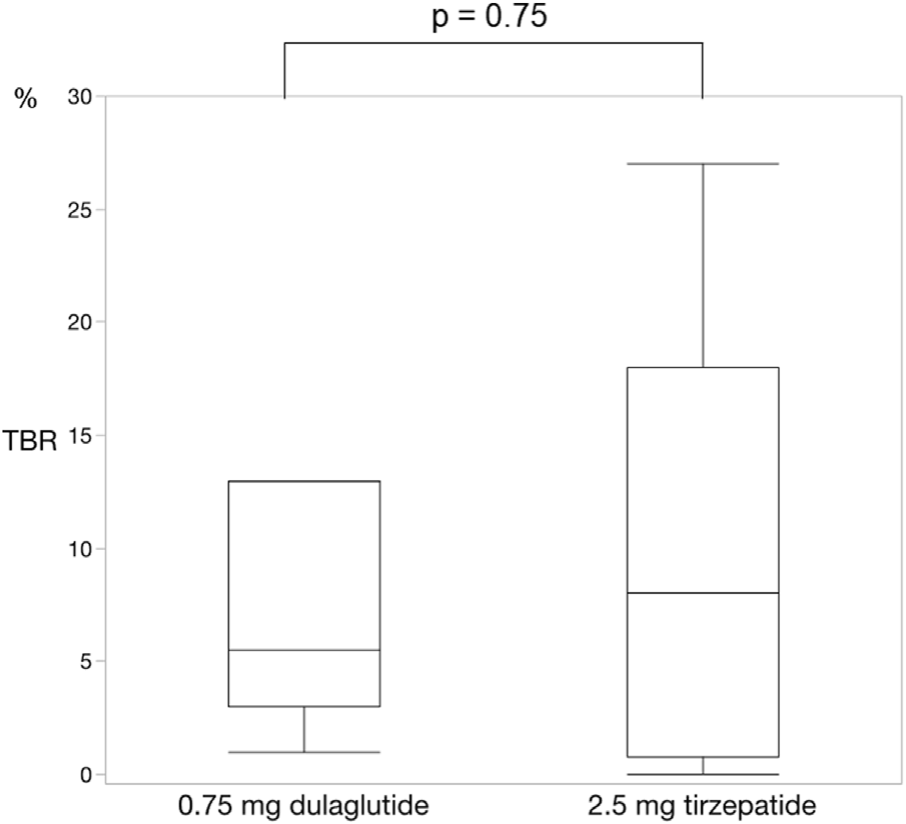
Change in time below range. There was no significant difference in the time below range between dulaglutide and tirzepatide. Wilcoxon rank-sum test was used in the analysis. TBR, time below range.

**FIGURE 6:**
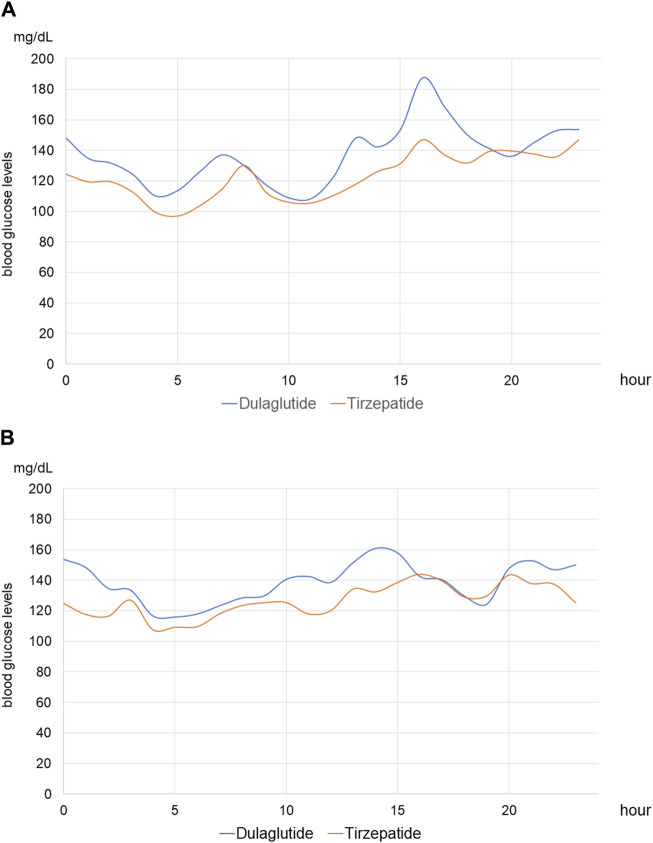
Median continuous blood glucose levels on hemodialysis and non-hemodialysis days **(A)** Median continuous blood glucose levels of all patients on hemodialysis days. The blue line shows those in periods of treatment with dulaglutide, and the red line shows those in periods of treatment with tirzepatide. **(B)** Median continuous blood glucose levels of all patients on non-hemodialysis days.

**FIGURE 7:**
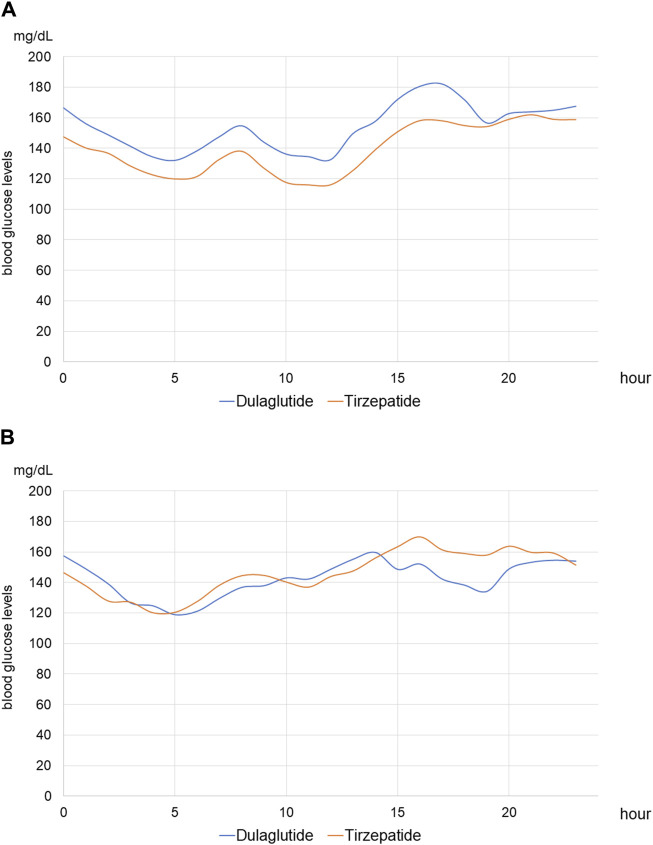
Average blood glucose level on hemodialysis and non-hemodialysis days of continuous glucose monitoring **(A)** Average continuous blood glucose levels of all patients on hemodialysis days. **(B)** Average continuous blood glucose levels of all patients on non-hemodialysis days.

For each of the 14 patients, CGM was carried out during 3 dialysis days and 4 non-dialysis days before and after switching to tirzepatide, resulting in CGM data being collected for a total of 42 dialysis days and 56 non-dialysis days. Before switching to tirzepatide, TIR, TAR, and TBR values were 42.7%, 48.4%, and 8.9%, respectively. The mean glucose level was 156.6 mg/dL. After switching to tirzepatide, the TIR increased to 50.8% (*p* = 0.02) (Figure 2), while the TAR and mean glucose level decreased to 37.8% (*p* = 0.02) (Figure 3) and 137.4 mg/dL (*p* = 0.006) (Figure 4), respectively. In contrast, the TBR was 11.3%, and there was no significant difference in the TBR between the baseline and after tirzepatide administration (*p* = 0.75) (Figure 5). TIR, TAR, TBR, and mean glucose levels were also analyzed separately on hemodialysis days and non-hemodialysis days. On hemodialysis days, before and after switching to tirzepatide, TIR were 44.8% and 52.2% (*p* = 0.02), TAR were 40.9% and 32.6% (0.07), TBR were 13.7% and 14.3% (*p* = 0.76), and mean glucose levels were 144.0 mg/dL and 130.5 mg/dL (*p* = 0.02), respectively. (Supplementary Figure S1) On non-hemodialysis days, TIR were 47.9% and 51.7% (*p* = 0.25), TAR were 46.9% and 35.4% (*p* = 0.03), TBR were 11.6% and 8.4% (*p* = 0.37), and mean glucose levels were 157.9 mg/dL and 141.3 mg/dL (*p* = 0.08), respectively. (Supplementary Figure S2) The median and the average of the continuous blood glucose levels plots of all the included patients on hemodialysis days and non-dialysis days are shown in Figures 6A, B, 7A, B, respectively. The differences in blood glucose levels from the baseline on hemodialysis days and non-hemodialysis days are shown in Supplementary Figures S3A, B, S4A, B, respectively.

Three months after switching to tirzepatide, glycemic albumin did not show significant changes compared to that before switching (21.4 ± 3.6% to 20.6 ± 5.1%), and dry weight decreased from 71.6 kg to 70.1 kg (−1.5 ± 0.3 kg, *p* = 0.001). Dyspepsia was observed in three patients and nausea was observed in one patient shortly after switching to tirzepatide, however; there were no critical adverse events reported for 3 months (Table 2).

**TABLE 2:** Adverse events after transitioning from dulaglutide to tirzepatide.

Event	No. of patients (%)
Death	0 (0)
Nausea	1 (7.1)
Vomiting	0 (0)
Diarrhea	0 (0)
Dyspepsia	3 (21.4)

Data expressed as the number of patients (%).

## 4 Discussion

This retrospective study analyzed the serum glucose levels in switching from dulaglutide to tirzepatide in patients with type 2 diabetes undergoing hemodialysis using CGM.

A previous study on patients with type 2 diabetes who did not undergo hemodialysis revealed robust improvements in glycemic control and body weight without an increased risk of hypoglycemia. Participants were randomly assigned to four groups (1:1:1:1): one group received tirzepatide once a week at 5 mg, another group at 10 mg, a third group at 15 mg, and the fourth group received a placebo. They were then monitored for 40 weeks. The average reduction in hemoglobin A1c (HbA1c) levels from the starting point was 1.87% for those on tirzepatide 5 mg, 1.89% for those on tirzepatide 10 mg, and 2.07% for those on tirzepatide 15 mg, in contrast to an increase of 0.04% for those on placebo. This led to estimated differences in treatment effectiveness compared with placebo of −1.91% for tirzepatide 5 mg, −1.93% for tirzepatide 10 mg, and −2.11% for tirzepatide 15 mg (all *p* < 0.001). Tirzepatide induced a dose-dependent reduction in body weight of 7.0–9.5 kg (Rosenstock et al., 2021). Similarly, tirzepatide improved glycemic control compared with semaglutide and insulin degludec (Frías et al., 2021; Ludvik et al., 2021). The SURPASS-4 study found that tirzepatide was more effective than glargine in reducing HbA1c levels in patients with type 2 diabetes and high cardiovascular risk. Additionally, it resulted in fewer cases of hypoglycemia, which can raise the risk of cardiovascular events (Del Prato et al., 2021). However, there is limited information on how well tirzepatide works in type 2 diabetes patients who are on hemodialysis.

Dulaglutide improved glycemic control without inducing hypoglycemia in type 2 diabetes undergoing hemodialysis (Yajima et al., 2018; Ugamura et al., 2022). Moreover, glycoalbumin levels (median −1.8%; *p* = 0.026) and the daily total insulin dose (−15.0 U/day; *p* = 0.002) significantly decrease (Ugamura et al., 2022). The mean and % CV of glucose levels significantly decrease after dulaglutide administration according to CGM (Yajima et al., 2018). Although the GLP-1RA dulaglutide is used in patients undergoing hemodialysis, some patients experienced inadequate glycemic control with dulaglutide.

In the general population, tirzepatide is superior to dulaglutide in terms of glycemic control (Inagaki et al., 2022; Nicholls et al., 2024). The use of tirzepatide in patients with chronic kidney disease (CKD) depends on three aspects. The first is the safety of GLP-1 in patients with CKD as well as the general population. A previous study showed that renal impairment did not have any clinically relevant effect on tirzepatide pharmacokinetics (Urva et al., 2020). The second is the efficacy of GLP-1 in patients with CKD as well as the general population. A previous study reported that tirzepatide is also effective in glycemic control in patients including those with CKD (Del Prato et al., 2021; Nicholls et al., 2024). The third is the protection of renal function. Tirzepatide treatment reduced albuminuria and the estimated glomerular filtration rate (eGFR) slope in patients with type 2 diabetes having CKD (Heerspink et al., 2022; Bosch et al., 2023). Recently, the renoprotective effects of tirzepatide were demonstrated using cystatin C-derived eGFR (Heerspink et al., 2023). In patients with type 2 diabetes and at least moderately increased albuminuria, a combination treatment of SGLT2 inhibitors, GLP-1 RA, and a nonsteroidal mineralocorticoid receptor antagonist resulted in observed gains in cardiovascular and kidney event-free and overall survival (Neuen et al., 2024). Thus, tirzepatide use seems to be reasonable in patients with CKD.

However, the efficacy of tirzepatide compared to dulaglutide in patients undergoing hemodialysis remain unclear. Thus, using CGM, this retrospective study analyzed the effect of switching from dulaglutide to tirzepatide on serum glucose levels in patients with type 2 diabetes undergoing hemodialysis.

Recommendations from the International Consensus on Time In Range state that the target range of TIR is over 70% per day (Battelino et al., 2019). However, the TIR during the period of once-weekly dulaglutide was only 42.7% in our study, indicating that additional anti-diabetic agents should be considered to achieve the target range of TIR. Furthermore, international consensus guidelines specify a target range for TBR of <4% and <1% in patients aged 60 years or those at high risk (Battelino et al., 2019). This study observed a TBR of 8.9% during the dulaglutide administration period, even in the absence of symptomatic hypoglycemia. This TBR level was significantly higher in high-risk patients, and alternative anti-diabetic agents are preferable to dulaglutide to decrease hyperglycemia and hypoglycemia.

Tirzepatide is a dual GLP-1 and GIP agonist, not solely a GLP-1RA. It may achieve more favorable glycemic control than dulaglutide through three possible mechanisms. First, GLP-1 and GIP interact with each other to improve blood glucose levels more effectively compared with GLP-1 single agonists. GLP-1RA acts synergistically with GIP activation to gain a broad improvement in metabolic health with the hypothesis that enhancing insulin secretion by dual actions on pancreatic β cells improves glycemia, restores sensitivity to GIP, and involves additional mechanisms of action (Zhou et al., 2023). Second, tirzepatide could decrease blood glucose levels more effectively in the hyperglycemic state, while not affecting glucose levels in normal and/or hypoglycemic states. This is because glucagon levels are based on blood glucose levels. In contrast to GLP-1, GIP exhibits glucagonotropic effects in normal and/or hypoglycemic states; conversely, it suppresses glucagon secretion in the hyperglycemic state (Christensen et al., 2011). Moreover, GLP-1 receptor expression decreases in a hyperglycemic state, but GIP receptor expression increases under the effect of acute hyperglycemia (Willard et al., 2020). Our CGM plots also showed reduced fluctuations in blood glucose levels after switching from dulaglutide to tirzepatide. In particular, post-dialysis blood glucose levels were elevated when treated with dulaglutide, whereas this elevation was suppressed after switching to tirzepatide. On the other hand, CGM plots on non-dialysis days did not show an obvious improvement. The improvement of glycemic control might be due to not on the non-hemodialysis days but on the hemodialysis days, and a longer-term study is needed to elucidate the efficacy of tirzepatide. Third, tirzepatide is an imbalanced dual agonist that favors the GIP receptor over GLP-1, and it is preferable to maximize glycemic control while suppressing gastrointestinal disorders (Finan et al., 2013). GLP-1 has multiple glucose-lowering actions, one of which is delaying gastric emptying. This effect induces gastrointestinal disorders, such as nausea and vomiting, which makes it difficult to increase the dose of GLP-1RA. However, this effect was not described for GIP. Thus, an imbalanced dual agonist favoring the GIP receptor over GLP-1 can achieve better glycemic control than GLP-1RA alone.

The most common adverse events in the patients administered tirzepatide or dulaglutide were nasopharyngitis (tirzepatide range 13.8–18.2% vs. dulaglutide 16.4%), nausea (11.9–20.0% vs. 7.5%), and constipation (13.8–17.7% vs. 10.7%) (Inagaki et al., 2022). In our study, dyspepsia and nausea were observed at 21.4% and 7.1%, respectively, which were slightly higher than the prior observation; however, nasopharyngitis and constipation were not observed, and no patient needed to stop the treatment owing to adverse events, with no critical adverse events reported.

In our study, dry weight decreased by approximately 1.5 kg after 3 months of transitioning to tirzepatide. All tirzepatide doses are superior to all comparators in terms of body weight reduction (Jastreboff et al., 2022; Karagiannis et al., 2022). This is probably because tirzepatide has the same effect on gastric emptying delay as selective GLP-1RA (Urva et al., 2020). Moreover, GIP increases lipogenesis, and enhances the lipid-buffering capacity of white adipose tissue (Samms et al., 2020). These mechanisms seem to improve obesity. In patients with early-stage CKD, a body mass index ≥35 kg/m^2^ is associated with poorer outcomes in terms of renal function (Lu et al., 2014). However, the relationship between obesity and CKD progression is still controversial. Moreover, some reports show that obesity is associated with improved survival in patients undergoing hemodialysis (Schmidt and Salahudeen, 2007). Recently, the presence of both sarcopenia and obesity, termed sarcopenic obesity, has been considered to be a risk factor for mortality and cardiovascular diseases in patients undergoing hemodialysis (de Oliveira Matos et al., 2022). Tirzepatide will improve obesity, but whether it also reduces the mortality in patients undergoing hemodialysis remains controversial.

Our facility routinely examines glycoalbumin instead of HbA1cas a glycemic control parameter. The standard method of monitoring glycemic control has been the periodic measurement of the level of HbA1c (Davies et al., 2018). However, HbA1c is influenced by various factors in patients undergoing hemodialysis, such as shortened erythrocyte lifespan, administration of erythropoietin as a stimulating agent for the treatment of renal anemia, the administration of iron preparations, uremia, and blood transfusion, all of which have the potential for rendering HbA1c measurements inaccurate (Abe et al., 2022). Glycoalbumin is more strongly correlated with plasma glucose levels than HbA1c in patients undergoing hemodialysis (Chen et al., 2017; Hoshino et al., 2018; Kohzuma et al., 2021). Nonetheless, glycoalbumin reflects average glucose levels and not fluctuating blood glucose levels. In patients undergoing hemodialysis, blood glucose levels tend to fluctuate, with frequent large glycemic excursions; therefore, it is crucial to use parameters that reflect fluctuations in blood glucose levels. CGM sensors continuously measure glucose concentrations in the interstitial fluid using a glucose oxidase reaction. Thus, CGM can track the fluctuation in blood glucose levels, thereby helping to provide accurate glycemic control and prevent hypoglycemia (Galindo et al., 2023). We believe that CGM is the most reliable method for effective glycemic control in patients undergoing hemodialysis.

This study has several limitations. First, the sample size was relatively small. Second, we used CGM and measured glycoalbumin, although we did not measure HbA1c. Continuous glucose monitoring reflects glycemic variability more accurately; however, it cannot be compared with other studies using HbA1c. Third, according to CGM data separated into hemodialysis and non-dialysis days, blood glucose levels improved substantially on hemodialysis days; however, on non-dialysis days there was no obvious improvement. Finally, the observation period was short, and a prolonged observation period is needed to clarify the long-term effectiveness and adverse events of tirzepatide in patients undergoing hemodialysis.

## 5 Conclusion

In our study, TIR increased without an increase in hypoglycemic episodes after switching from dulaglutide to tirzepatide in patients with type 2 diabetes undergoing hemodialysis. Transitioning from dulaglutide to tirzepatide can improve glycemic control without increasing hypoglycemia in patients undergoing hemodialysis for type 2 diabetes. A large-scale study is required to verify these results.

## Data Availability

The raw data supporting the conclusion of this article will be made available by the authors, without undue reservation.

## Supplementary material

SUPPLEMENTARY FIGURE S1 Change of time in range, time above range, time below range, and mean blood glucose levels on hemodialysis days. The time in range and the mean blood glucose levels improved upon changing from dulaglutide to tirzepatide on hemodialysis days. Wilcoxon rank-sum test was used in the analysis. **(A)** Change of time in range on hemodialysis days **(B)** Change of time above range on hemodialysis days **(C)** Change of time below range on hemodialysis days **(D)** Change of mean blood glucose levels on hemodialysis days.

SUPPLEMENTARY FIGURE S2 Change of time in range, time above range, time below range, and mean blood glucose levels on non-hemodialysis days. The time in range did not show significant improvement upon changing from dulaglutide to tirzepatide on non-hemodialysis days. The time above range improved changing from dulaglutide to tirzepatide on hemodialysis days. Wilcoxon rank-sum test was used in the analysis. **(A)** Change of time in range on non-hemodialysis days **(B)** Change of time above range on non-hemodialysis days **(C)** Change of time below range on non-hemodialysis days **(D)** Change of mean blood glucose levels on non-hemodialysis days.

SUPPLEMENTARY FIGURE S3 The differences in median blood glucose levels from the baseline on hemodialysis and non-hemodialysis days of continuous glucose monitoring. The differences in median blood glucose levels of all patients from the baseline on hemodialysis are shown, and the baselines are the time of starting CGM. The blue line shows those in periods of dulaglutide treatment, and the red line shows those in periods of tirzepatide treatment. **(A)** The difference in median blood glucose levels from the baseline on hemodialysis **(B)** The difference in median blood glucose levels from the baseline on non-hemodialysis.

SUPPLEMENTAL FIGURE S4 The differences in average blood glucose levels from the baseline on hemodialysis and non-hemodialysis days of continuous glucose monitoring. The differences in average blood glucose levels of all patients from the baseline on hemodialysis are shown, and the baselines are the time of starting CGM. The blue line shows the average blood glucose levels during dulaglutide treatment, and the red line shows those during tirzepatide treatment. **(A)** The difference in average blood glucose levels from the baseline on hemodialysis **(B)** The difference in average blood glucose levels from the baseline on non-hemodialysis.
