# Supplementary figures and images for: Improving glycemic control: transitioning from dulaglutide to tirzepatide in patients with type 2 diabetes undergoing hemodialysis
Source: Front Pharmacol. 2024 May 30;15:1362242. doi: 10.3389/fphar.2024.1362242 (PMC11169618; doi:10.3389/fphar.2024.1362242)

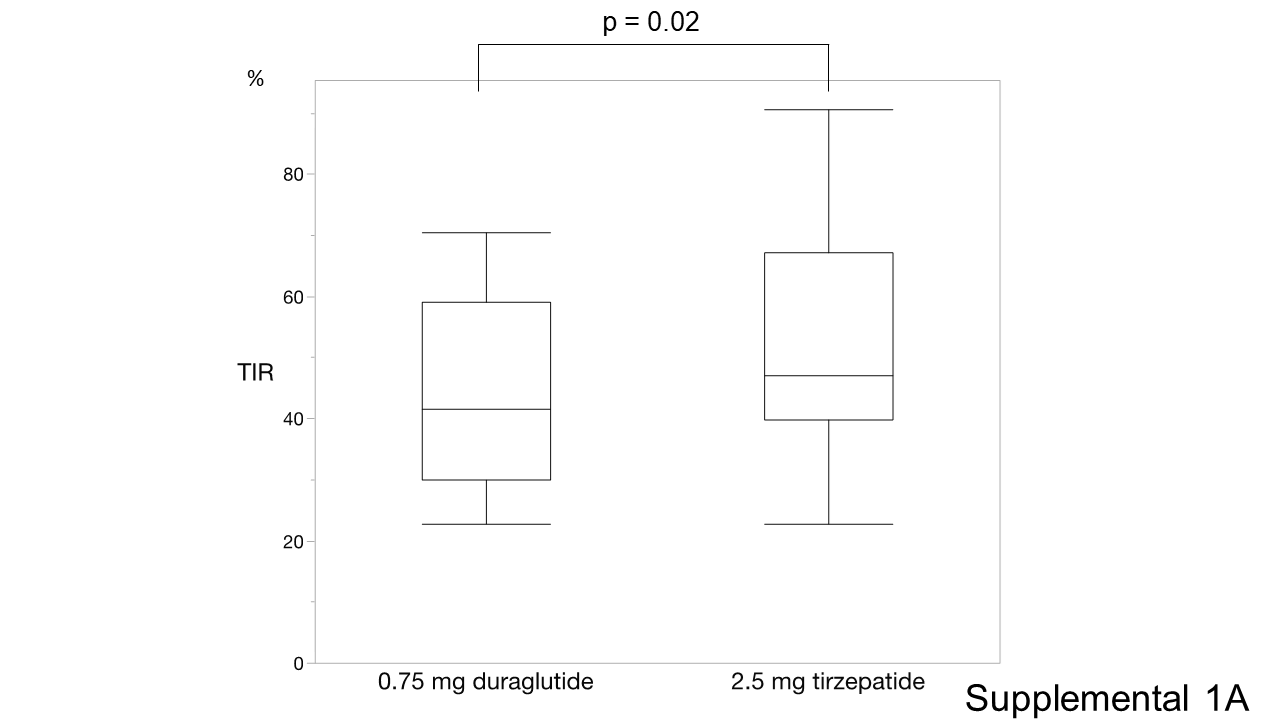

Supplement: Supplementary file 1 [file DataSheet1.zip › Image 1A.TIF]

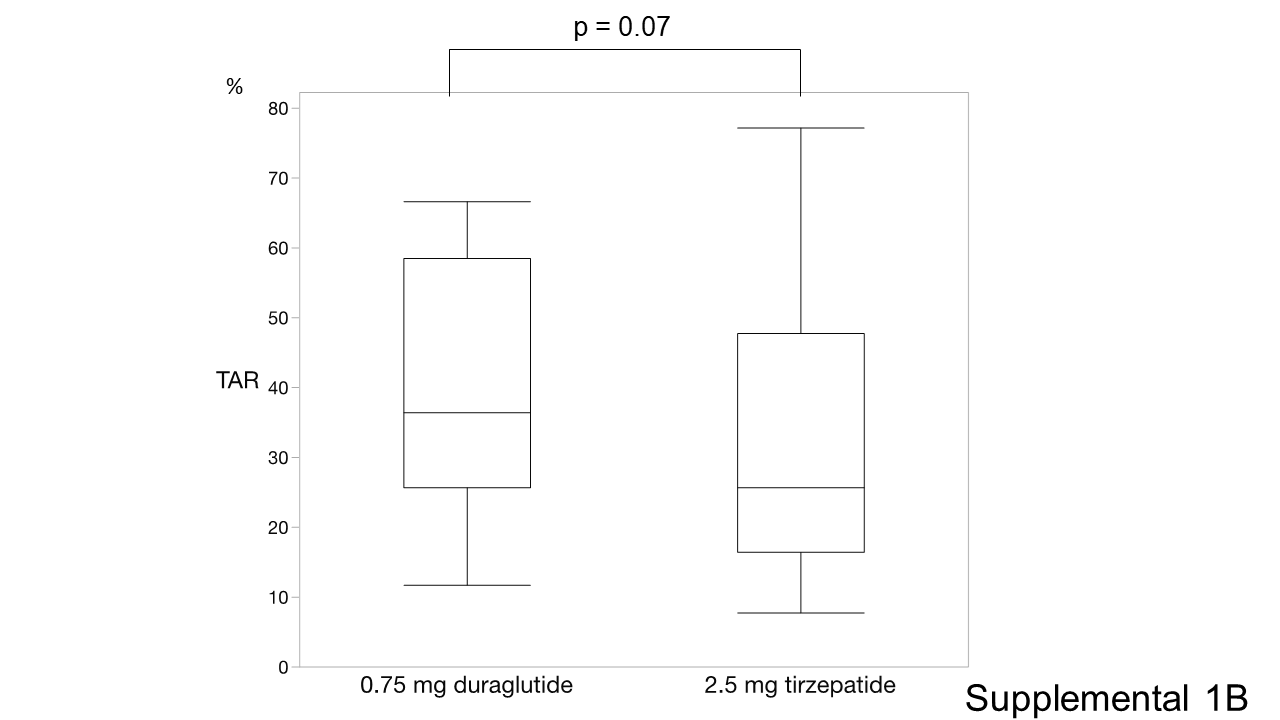

Supplement: Supplementary file 1 [file DataSheet1.zip › Image 1B.TIF]

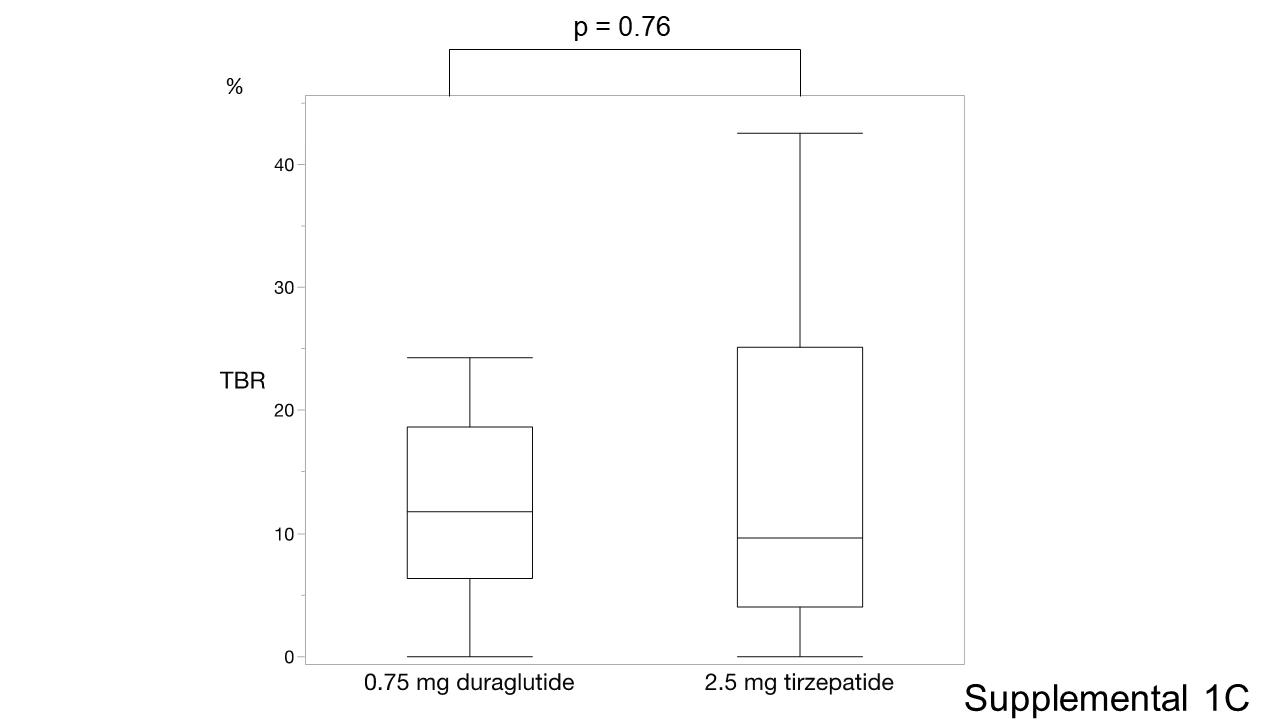

Supplement: Supplementary file 1 [file DataSheet1.zip › Image 1C.TIF]

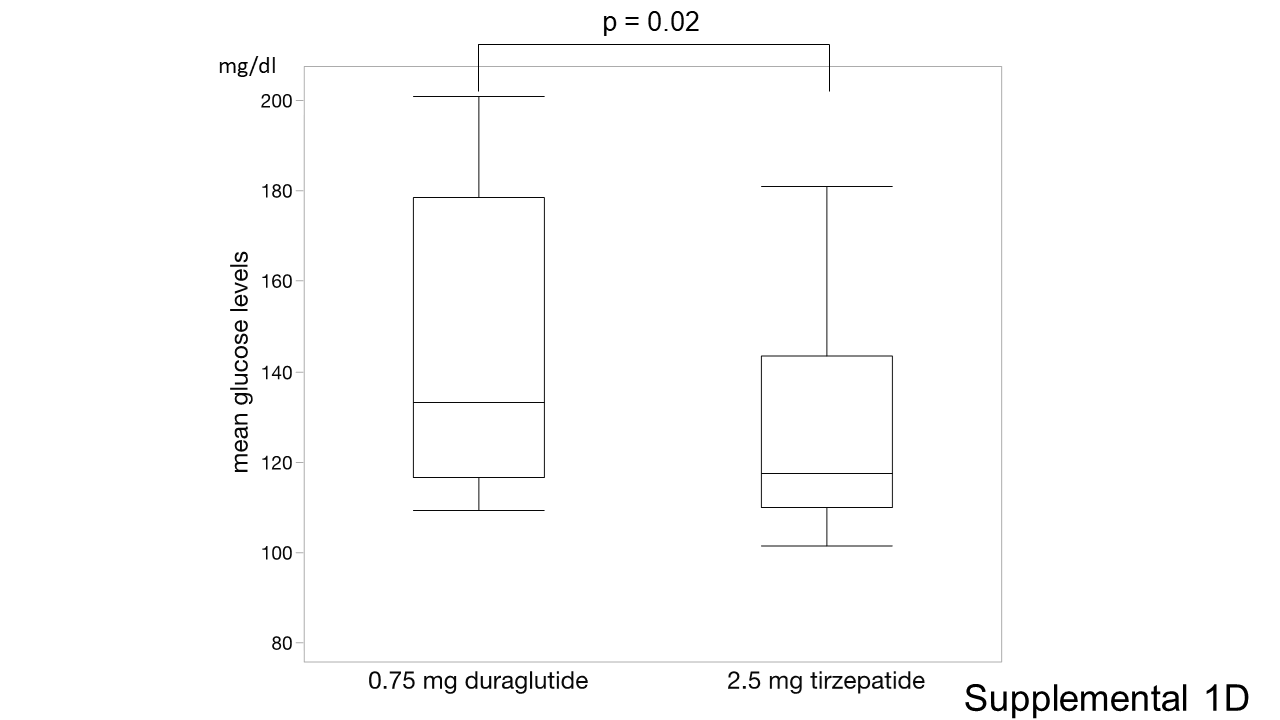

Supplement: Supplementary file 1 [file DataSheet1.zip › Image 1D.TIF]

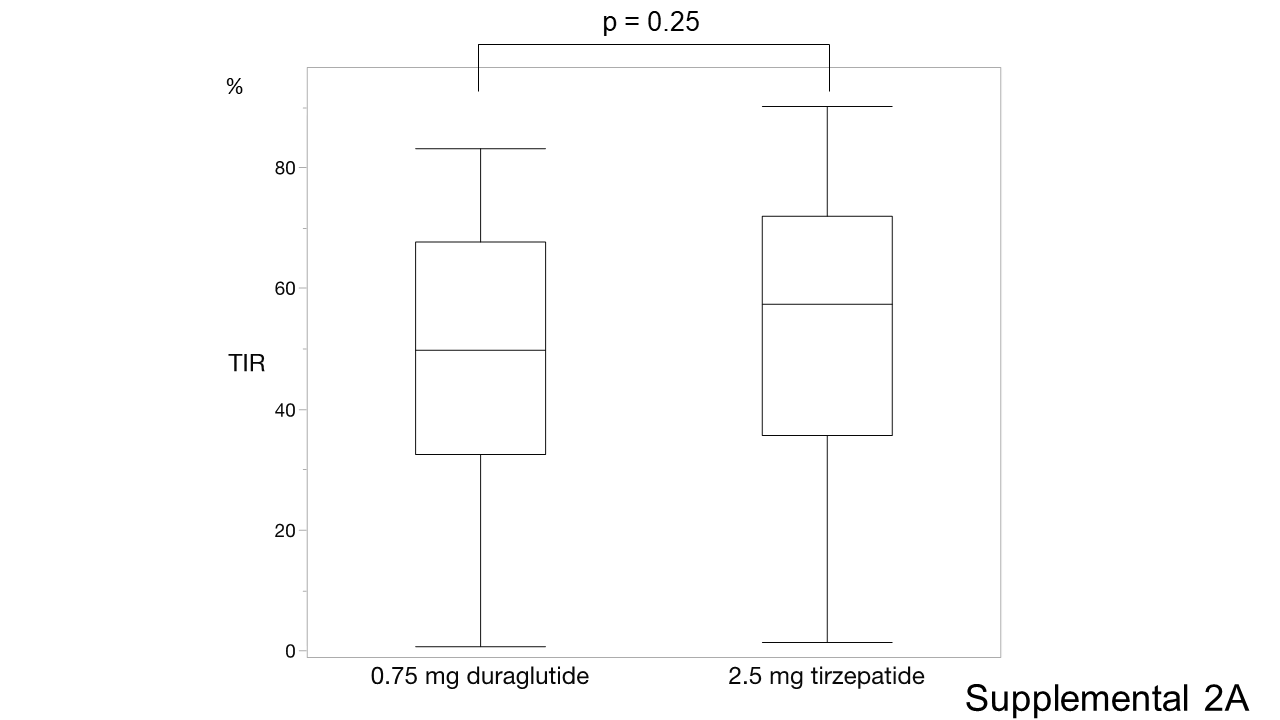

Supplement: Supplementary file 1 [file DataSheet1.zip › Image 2A.TIF]

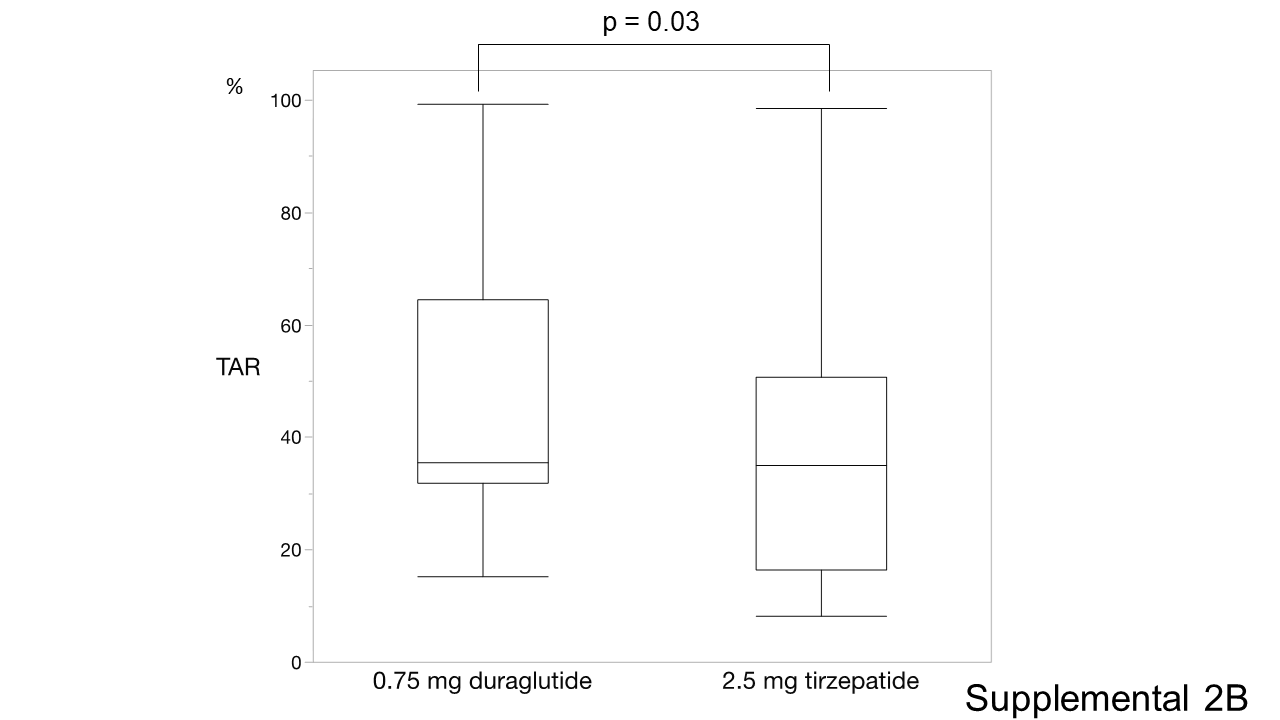

Supplement: Supplementary file 1 [file DataSheet1.zip › Image 2B.TIF]

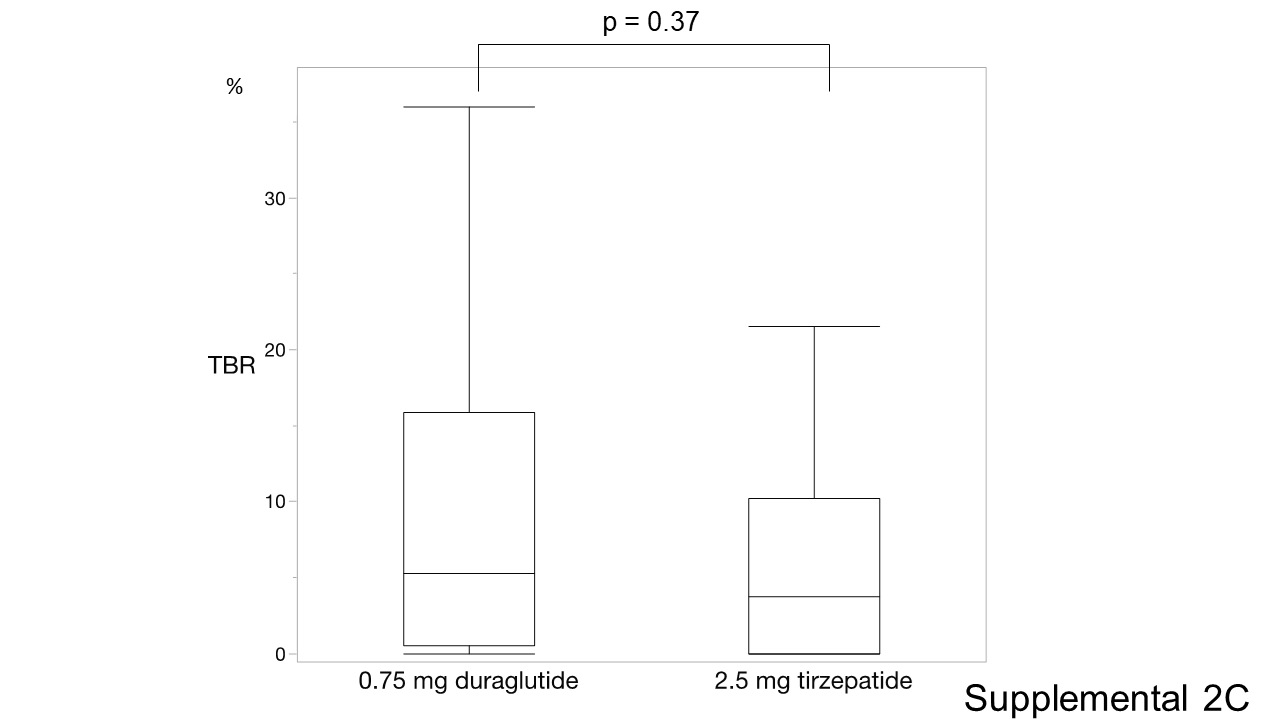

Supplement: Supplementary file 1 [file DataSheet1.zip › Image 2C.TIF]

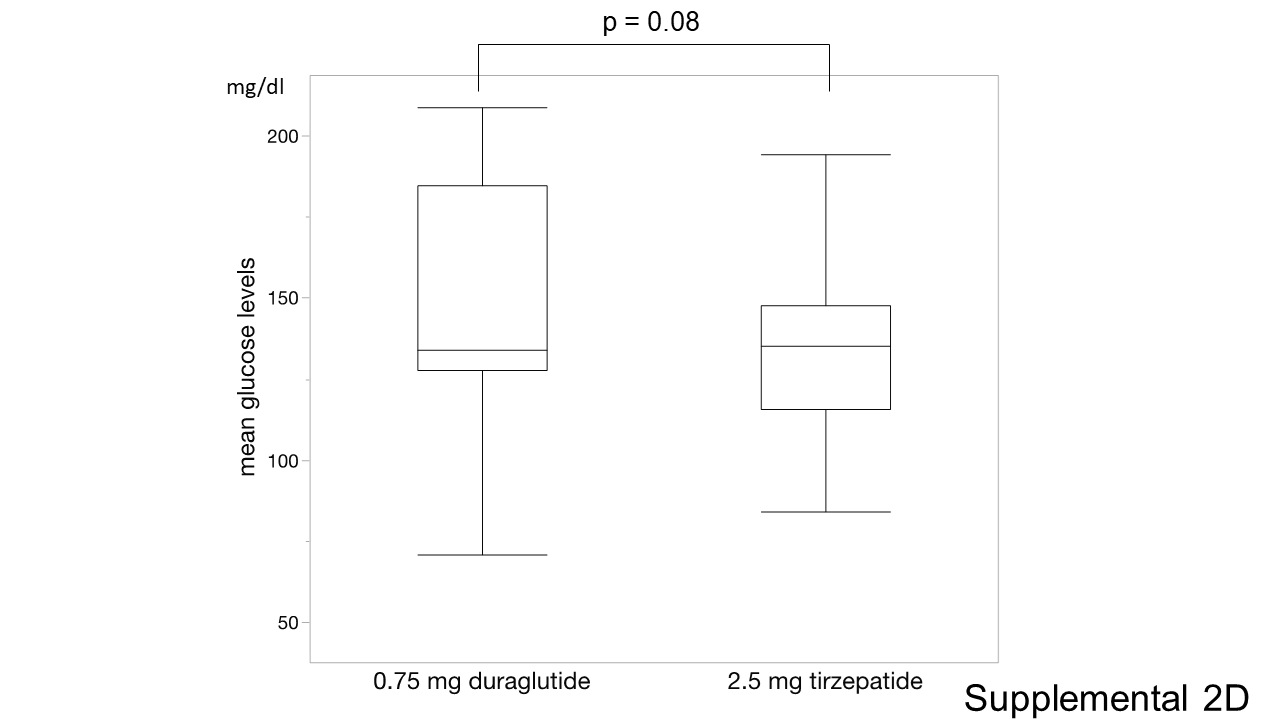

Supplement: Supplementary file 1 [file DataSheet1.zip › Image 2D.TIF]

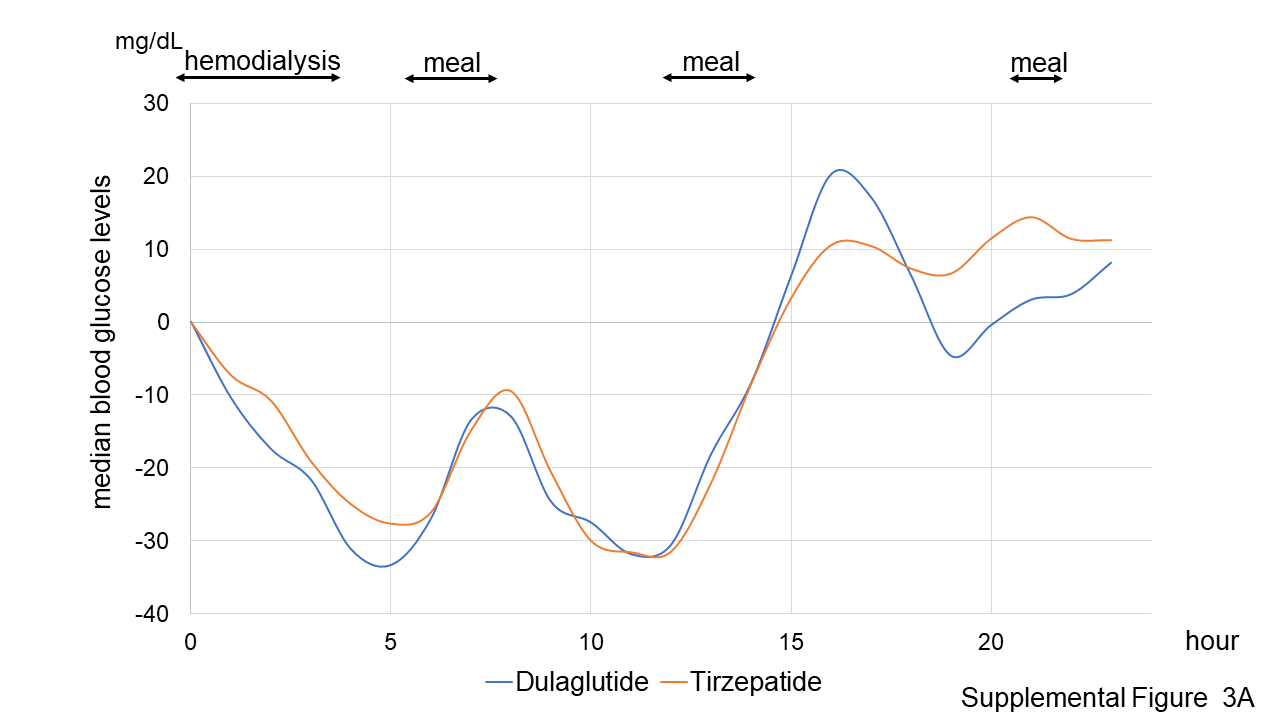

Supplement: Supplementary file 1 [file DataSheet1.zip › Image 3A.TIF]

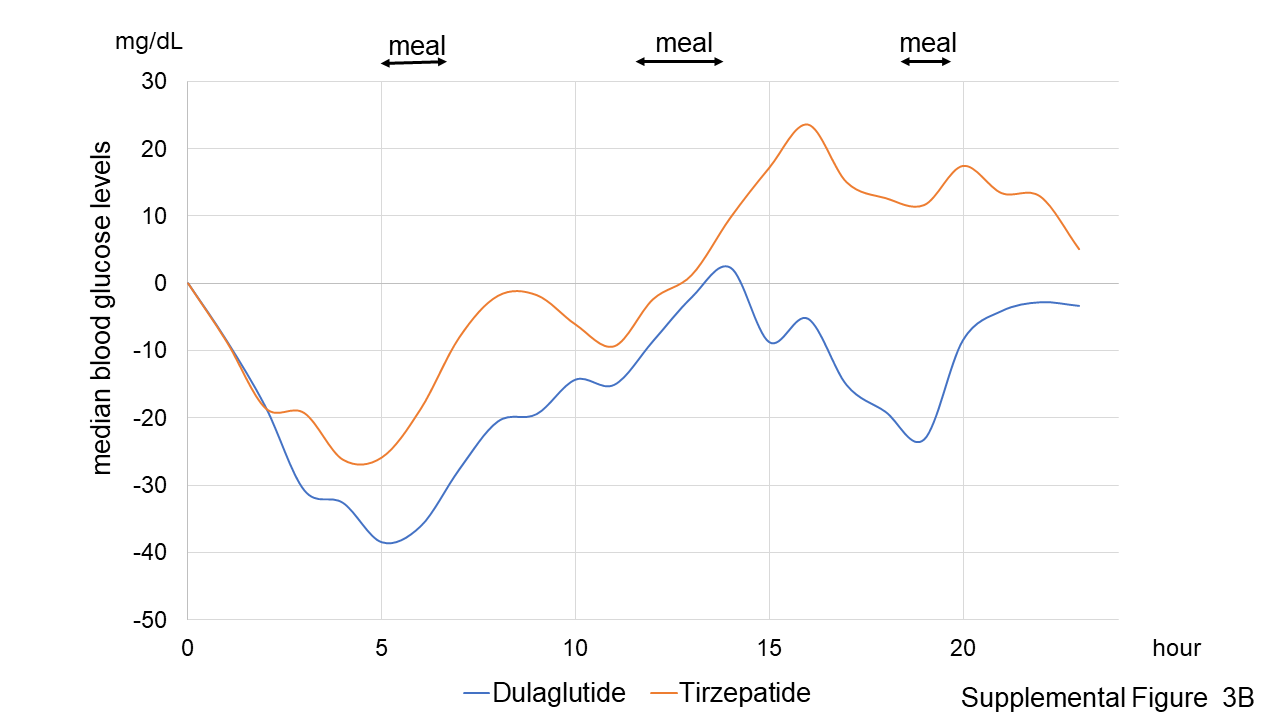

Supplement: Supplementary file 1 [file DataSheet1.zip › Image 3B.TIF]

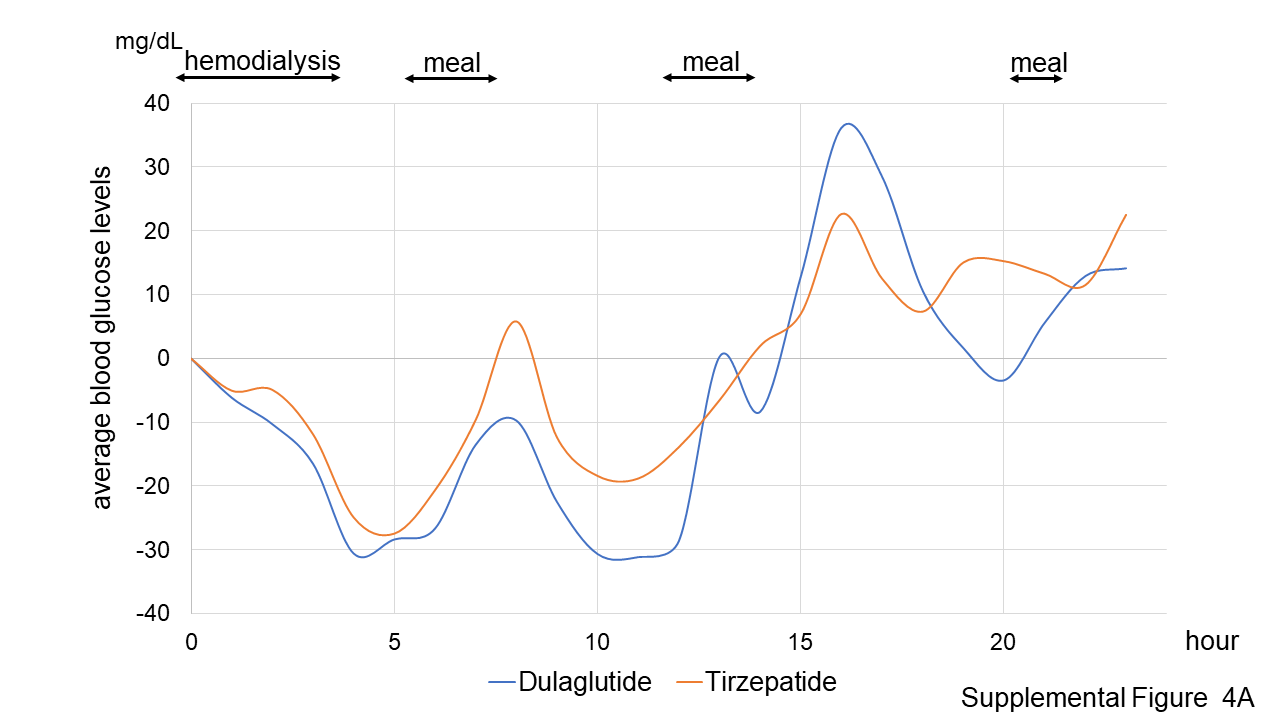

Supplement: Supplementary file 1 [file DataSheet1.zip › Image 4A.TIF]

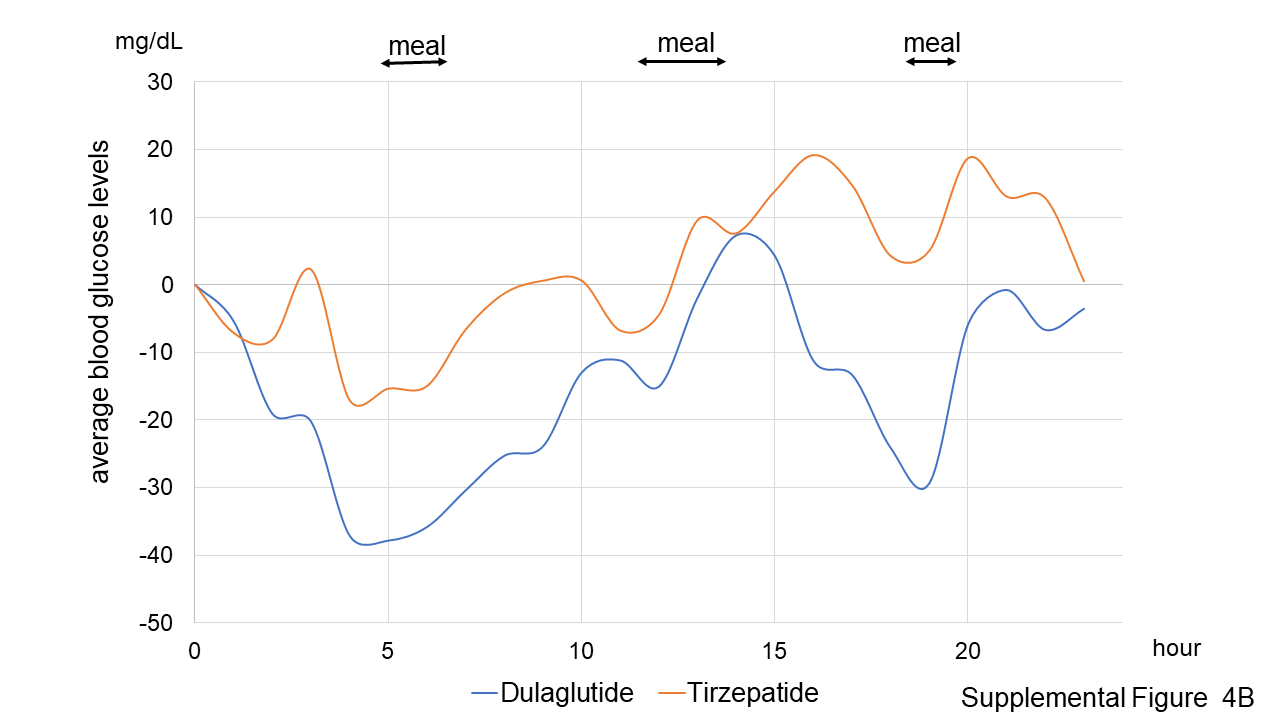

Supplement: Supplementary file 1 [file DataSheet1.zip › Image 4B.TIF]
